# Integrating guideline development and implementation: analysis of guideline development manual instructions for generating implementation advice

**DOI:** 10.1186/1748-5908-7-67

**Published:** 2012-07-23

**Authors:** Anna R Gagliardi, Melissa C Brouwers

**Affiliations:** 1 University Health Network, 200 Elizabeth Street, Toronto, Ontario, Canada; 2 McMaster University, Juravinski Hospital Site, 711 Concession Street, Hamilton, Ontario, Canada

**Keywords:** Guideline development, Guideline implementation, Implementability

## Abstract

**Background:**

Guidelines are important tools that inform healthcare delivery based on best available research evidence. Guideline use is in part based on quality of the guidelines, which includes advice for implementation and has been shown to vary. Others hypothesized this is due to limited instructions in guideline development manuals. The purpose of this study was to examine manual instructions for implementation advice.

**Methods:**

We used a directed and summative content analysis approach based on an established framework of guideline implementability. Six manuals identified by another research group were examined to enumerate implementability domains and elements.

**Results:**

Manuals were similar in content but lacked sufficient detail in particular domains. Most frequently this was Accomodation, which includes information that would help guideline users anticipate and/or overcome organizational and system level barriers. In more than one manual, information was also lacking for Communicability, information that would educate patients or facilitate their involvement in shared decision making, and Applicability, or clinical parameters to help clinicians tailor recommendations for individual patients.

**Discussion:**

Most manuals that direct guideline development lack complete information about incorporating implementation advice. These findings can be used by those who developed the manuals to consider expanding their content in these domains. It can also be used by guideline developers as they plan the content and implementation of their guidelines so that the two are integrated. New approaches for guideline development and implementation may need to be developed. Use of guidelines might be improved if they included implementation advice, but this must be evaluated through ongoing research.

## Background

Research, practice, and policy in the healthcare sector focus on improving the organization, delivery, and outcomes of care. Critical to achieving these objectives is the need for guidance based on currently available knowledge generated through research. This has led to an emphasis on evidence syntheses such as guidelines that are defined as ‘systematically developed statements to assist practitioner and patient decisions about appropriate healthcare for specific clinical circumstances’ [1,2]. While guidelines are important decision-making tools, along with expert clinical judgment and patient preference, their impact remains variable. Perhaps the most convincing evidence of this comes from a population-based assessment of performance on 439 recommendations for 30 conditions spanning preventive, acute, and chronic services that found that only 55% of patients in the United States received recommended care [3]. Similar lack of adherence to practice guidelines continues to be identified worldwide across different conditions and settings of care [4-7].

Numerous factors influence whether and how guidelines are used. Some of these are extrinsic factors, such as: the nature of the newly recommended practice or technology itself; characteristics of healthcare providers; organizational capacity to collect, adapt, share, and apply evidence; and system-level environmental factors [8-10]. Because these manifest downstream of guideline development, single and combined interventions can be applied to address these barriers and improve compliance with guideline recommendations, although their impact can be variable and inconsistent [11]. Other factors are intrinsic to guidelines and perhaps best addressed at the time of guideline development. For example, guidelines may be biased through conflict of interest, of variable methodological quality, poorly written and ambiguously presented, viewed as not applicable to individual patients or reducing clinician autonomy, and the volume of guidelines now available may be overwhelming, particularly given that recommendations for the same clinical indication may be inconsistent across different guidelines [12-14].

To promote the development and use of high quality guidelines, the Appraisal of Guidelines Research and Evaluation (AGREE) instrument was developed by an international collaboration of guideline development experts [15]. A revised version, AGREE II, was recently issued [16]. It can be used to score guidelines based on their reported scope and purpose, stakeholder involvement, rigour of development, clarity of presentation, editorial independence, and applicability. The Guideline Implementability Appraisal (GLIA) instrument is another tool that similarly considers the impact of guideline recommendations on individuals and organizations, and whether measurable indicators are provided by which to evaluate implementation [17]. Still, many guidelines fail to comply with these recommended standards. Shaneyfelt *et al*. evaluated 279 guidelines published from 1985 to June 1997 by 69 different developers, and found that mean overall adherence to standards for guideline format, methods, identification and summary of evidence, and formulation of recommendations was 43% [18]. When the AGREE instrument was used to evaluate 86 guidelines from 11 countries published between 1992 and 1999, they were found to vary considerably in whether and how they addressed each domain [19].

Turner *et al.* proposed that variability in manuals describing guideline development methods may be causing the mismatch between standards for guideline quality and the actual quality of guidelines [20]. They identified and examined six prominent guideline development manuals and found strong concordance in recommended guideline development methods across all manuals. However, the instructions varied in level of detail, so the authors hypothesized that manuals may not provide sufficient guidance on how to complete all steps of the guideline development process.

It is now well recognized that simply producing guidelines does not itself lead to use, and that active implementation is needed to encourage their uptake. The Knowledge to Action Cycle offers a framework for promoting the use of guidelines [21]. It recommends an iterative process comprised of several steps, including adapting guidelines to local context, identifying barriers of guideline use, selecting and implementing tailored interventions to promote guideline use, monitoring guideline use, evaluating outcomes associated with guideline use, and sustaining guideline use. Regardless of whether guideline developers are mandated and resourced to implement the guidelines they produce, or whether it is the responsibility of other individuals or organizations to implement guidelines, detailed instructions for guideline implementation are needed. Turner *et al*. found that four of six manuals described instructions for developing implementation advice that could be included in guidelines. However, the framework they used to examine manuals was based largely on the AGREE instrument, supplemented by a literature search for other key methods related to the development of guidelines. The framework consisted of 14 elements organized in four domains: preparing for guideline development, systematically reviewing the evidence, drafting the guideline, and reviewing the guideline. Only one element pertained to implementation, and they only looked for presence or absence of any implementation information, and not the details of instructions for developing implementation advice.

We investigated how to make guidelines more implementable by modifying their content and format [22]. We first conducted a review of the medical literature for features of guidelines desired by guideline users, or that are positively associated with guideline use. The framework was validated through review by health professionals and researchers with various clinical and disciplinary perspectives, and refined by using it to examine the content of published guidelines judged to be high quality by trained experts [22]. The final framework consisted of 22 elements within eight domains, including adaptability, usability, validity, applicability, communicability, accommodation, implementation, and evaluation (Table 1). Most guidelines we examined contained a large volume of graded evidence (validity) and numerous tables featuring complementary clinical information (applicability), but few contained additional features representing the other six domains that may facilitate application of guidelines by users. As proposed by Turner *et al*., guidelines may offer little support to users for implementing the recommendations because guideline development manuals lack instructions for developing such implementation advice. To confirm this and more thoroughly describe the nature of instructions for developing implementation advice in guideline development manuals, the purpose of this research was to re-examine the manuals sampled by Turner *et al*. according to our framework of implementability, which reflects guideline components shown by research to be associated with intent to, and actual use of guidelines. Use of a more detailed framework serves to pinpoint specific implementation information that may be lacking, thereby informing future updating of such manuals, or development of adjunct products.

**Table 1:** Guideline implementability framework

**Domain**	**Definition**	**Element**	**Examples**
Adaptability	The guideline is available in a variety of versions for different users or purposes	Sources	Internet, peer reviewed journal
		Versions	Full text, summary, print, digital
		Users	Tailored for patients or caregivers
Useability	Content is organized to enhancethe ease with which the guideline can be used	Navigation	Table of contents
		Evidence	Narrative, tabulated or both
		Recommendations	Narrative, graphic (algorithms) or both; Recommendation summary (single list in full or summary version rather than dispersed)
Validity	Evidence is summarized and presented such that its quantity and quality are apparent	Number of references	Total number of distinct references to evidence upon which recommendations are based
		Evidence graded	A system is used to categorize quality of evidence supporting each recommendation
		Number of recommendations	Total number of distinct recommendations
Applicability	Information is provided to help interpret and apply guidelines for individual patients	Clinical considerations	Information such as indications, criteria, risk factors, drug dosing that facilitates application of the recommendations explicitly highlighted as tips or practical issues using sub-titles or text boxes, or summarized in tables and referred to in recommendations or narrative
Communicability	Resources for providers or patients to inform, educate, support and involve patients	Inform, educate, support	Informational, educational or supportive resources for patients/caregivers, or contact information (phone, fax, email or URL) for such resources
		Decision making	Questions or tools for clinicians to facilitate discussion with patients, or decision aids to support patient involvement
Relevance	The focus or purpose of the guideline is explicitly stated	Objective	Explicitly stated purpose of guideline (clinical, education, policy, quality improvement)
		Stakeholders	Specify who would deliver (individuals, teams, departments, institutions, managers, policy makers, internal/external agents) and receive the services (specify type of patients)
		Needs	Identification of stakeholder needs, perspectives, interests or values
Accommodation	Anticipated changes, resources and competencies required to adapt and accommodate guideline utilization are identified	Technical	Equipment or technology needed, or the way services should be organized
		Regulatory	Industrial standards for equipment or technology, or policy regarding their use
		Human resources	Type and number of health professionals needed to deliver recommended services
		Professional	Education, training or competencies needed by clinicians/staff to deliver recommendations
		Workflow	Anticipated changes in workflow or processes during/after adoption of recommendations
		Costs	Direct or productivity costs incurred by acquiring resources or training to accommodate guidelines, or as a result of service reductions during transition from old to new processes
Implementation	Processes for planning and applying local strategies to promote guideline utilization are described	Identify barriers	Individual, organizational, or system barriers that could challenge adoption, or instructions for local needs assessment of guideline users
		Tailor guideline	Instructions, tools or templates to tailor guideline/recommendations for local context
		Integrated tools	Point-of-care templates/forms (clinical assessment, standard orders) to integrate guidelines within care delivery processes
		Promote utilization	Possible mechanisms by which to promote guideline utilization
Evaluation	Processes for evaluating guideline implementation and utilization are described	Implementation	Methods for evaluating the implementation process
		Utilization	Audit tools or performance measures/quality indicators to assess the organization, delivery and outcomes of guideline recommended care

## Methods

### Approach

Content analysis describes ideas in written, verbal, or visual communication to generate, extend, or validate a framework or model [23]. It involves preparation (select unit of analysis, review data), analysis (code, group text by category), and reporting (describe analysis, findings). There are three types of content analysis (conventional, directed, summative) that vary by inductive or deductive nature [24]. This study adopted a directed approach by examining the content of guideline development manuals based on domains and elements in the implementability framework, and a summative approach by synthesizing findings to compare the domains and elements included in each manual (summative). As with any qualitative research, the findings of content analysis can be quantified, for example, by counting the number of times a word is used or an idea expressed, or expressed more qualitatively by identifying themes and the way they are expressed [24]. We described the presence or absence of content reflecting implementability domains and elements quantitatively, and compared those findings across manuals.

### Data collection

The unit of analysis is the guideline development manual. These were sampled by convenience. We examined the same manuals identified by Turner *et al*. because they used a comprehensive strategy to search for and identify the manuals, including the MEDLINE database, various guideline web sites, web sites of known guideline developers, a general search of the Internet using Google, and consultation with members of an evidence-based healthcare email list. They included manuals published in English language, produced by international organizations responsible for guideline development, and supporting the development of evidence-based guidelines, and excluded manuals issued by specialty societies for specific conditions.

Full-text manuals were retrieved from organization web sites, which were checked once again at the time of writing this manuscript to ensure the most recent versions were analyzed. A data extraction form reflecting the implementability framework domains and elements was developed. It was used to extract information about the presence or absence of each element anywhere within the guideline development manuals, plus producer, date of publication, number of pages, stated objective of the manual, implementation context (the stated purpose or setting of implementation), and how the implementation content was organized within the manual. Two individuals independently extracted data, and a third resolved differences and tabulated the data.

### Data analysis

Tabulated findings were examined to describe general features of each manual, and enumerate the presence of implementation instructions reflecting elements from the implementability framework.

## Results

Manual features are summarized in Table 2. The six manuals were published between 1998 and 2011 by the Council of Europe (COE), National Health and Medical Research Council (NHMRC), National Institute for Health and Clinical Excellence (NICE), New Zealand Guidelines Group (NZGG), Scottish Intercollegiate Guideline Network (SIGN), and the World Health Organization (WHO) [25-30]. Their size ranged from 23 to 266 pages plus appendices. The stated objective of all manuals was similar: to inform guideline development. The stated implementation context was also similar across the six manuals, encompassing local patient care, regional management of services, and population-based policy setting. Five of the six manuals contained a specific chapter addressing implementation ranging from five to eleven pages [25-29], and one of the five offered a 104-page supplement specific to implementation [26].

**Table 2:** Features of guideline manuals

**Producer**	**Date published**	**Pages (#)**	**Manual objective**	**Implementation context**	**Section devoted specifically to guideline implementation**
COE (25)	2002	77	Make proposals on the methodology to be used in developing guidelines	Daily practice, Managerial, Patient, Legal, ethical	11 pages in chapter on Dissemination and Implementation
			Identify the practical, social, ethical and legal conditions for implementation of guidelines in daily practice		
NHMRC (26)	1999, 2000 (supplement)	79	Put forward a method for developing clinical practice guidelines in Australia	Local conditions (population, setting, costs, constraints, patient values/preferences)	9 pages in chapter on Dissemination and Implementation
			Reflect concern that greater emphasis should be placed on guideline implementation and evaluation		
NICE (27)	2009	266 plus appendix	Explain how NICE develops clinical guidelines	Patient care and patient outcomes in the National Health Service as a whole	6-page chapter
			Provide advice to guideline developers on technical aspects of guideline development and the methods used	Cost/resources	
NZGG (28)	2001	86 plus appendix	To be used by guideline development teams, to assist them to produce evidence-base clinical practice guidelines	Legislative, administrative, clinical, industrial, consumer	10-page chapter on Dissemination and Implementation with focus on implementation
SIGN (29)	2011	104	Provide a reference tool that may be used by individual members of guideline development groups as they work through the development process	Local National Health Service board	5-page chapter
			Allow users to see how SIGN guidelines are developed, and instill confidence … that the recommendations are both internally and externally valid, and feasible for practice		
WHO (30)	2008	23	Document the recommended approach to development of WHO guidelines	Global, public health	---

COE, Council of Europe; NHMRC, National Health and Medical Research Council (Australia); NICE, National Institute for Health and Clinical Excellence; NZGG, New Zealand Guidelines Group; SIGN, Scottish Intercollegiate Guidelines Network; WHO, World Health Organization.

Implementability domains and elements addressed in manuals are enumerated in Table 3. Most manuals contained some representation of the majority of these domains and elements, though detail was minimal and did not lend itself to thematic analysis. COE included all domains but Applicability, but few elements within the domains of Relevance and Accommodation [25]. NHMRC [26] included all domains in the main manual or implementation supplement but few elements in the domain of Accommodation, as did NICE [27]. NZGG did not include the domains of Applicability and Communicability, and few elements in the domain of Accommodation [28]. SIGN did not include the domain of Applicability, and few elements in the domains of Accommodation and Implementation [29]. WHO did not include the domains of Adaptability and Comunicability, few elements in the domain of Accommodation, and cursory mention of Implementation and Evaluation [30].

**Table 3:** Domains of implementability addressed in guideline manuals

**Domain/Element**	**Producer**						
	**COE**	**NHMRC**	**NICE**	**NZGG**	**SIGN**	**WHO**	**Total**
**Adaptability**							
Sources	✓	✓	✓	✓	✓	--	5
Versions	✓	✓	✓	✓	✓	--	5
Users	✓	✓	✓	✓	✓	--	5
**Usability**							
Navigation	--	--	✓	✓	--	--	2
Evidence	✓	--	✓	✓	✓	✓	5
Recommendations	✓	✓	✓	✓	✓	✓	6
**Validity**							
# references	--	--	--	--	--	--	0
Evidence graded	✓	✓	✓	✓	✓	✓	6
# recommendations	--	--	--	--	--	--	0
**Applicability**							
Individualization	--	✓	✓	--	--	✓	3
**Patient support**							
Patient resources	--	✓	✓	--	✓	--	3
Decision making	✓	--	--	--	✓	--	2
**Relevance**							
Objective	--	✓	✓	--	--	✓	3
Users	--	✓	✓	✓	✓	✓	5
Needs	✓	✓	✓	✓	✓	✓	6
**Accommodation**							
Technical	--	✓	--	--	--	--	1
Regulatory	--	--	--	--	--	--	0
Human resources	--	--	--	--	--	--	0
Professional	✓	✓	✓	--	✓	--	4
Workflow	✓	--	✓	--	✓	--	3
Costs	--	✓			--	✓	4
**Implementation**							
Identify barriers	✓	✓	✓	✓	✓	✓	6
Tailor recommendations	✓	✓	✓	--	--	✓	4
Integrated tools	✓	✓	✓	✓	--	✓	5
Strategies to promote use	✓	✓	✓	✓	✓	--	5
**Evaluation**							
Implementation	✓	✓	--	✓	✓	--	4
Utilization	✓	✓	✓	✓	✓	✓	6

COE, Council of Europe; NHMRC, National Health and Medical Research Council (Australia); NICE, National Institute for Health and Clinical Excellence; NZGG, New Zealand Guidelines Group; SIGN, Scottish Intercollegiate Guidelines Network; WHO, World Health Organization.

## Discussion

We examined the content of the same guideline development manuals reviewed by Turner *et al*. [20] using a more comprehensive framework to assess the degree to which they offered instructions for developing implementation advice. Our findings support the observation of Turner *et al*. that the manuals were similar but lacked sufficient detail. We did not identify trends in manual purpose, implementation context, or content by type of guideline developer. Use of a more comprehensive framework reflecting the multiple steps of implementation revealed specific topics not addressed. Most frequently this was Accomodation, or information that would help guideline users anticipate and/or overcome organizational- and system-level barriers. This may include: information about equipment or technology needed; industrial standards; policies governing their use; type and number of health professionals needed to deliver services; education, training, or competencies needed by staff to deliver services; anticipated changes in workflow or processes during or after adoption of guideline recommendations. In more than one manual, Communicability information was lacking, or resources that physicians and/or patients could use for education or to facilitate shared decision making, as was Applicability information, or clinical parameters to help physicians tailor guideline recommendations for individual patients.

While directions for creating Applicability information were absent in guideline development manuals, our previous work found that 90% of guidelines we examined did include clinical considerations by which to individualize recommendations [22]. This is likely because guidelines largely summarize data from clinical studies, and closely related to this is information such as diagnostic or risk criteria, pharmacologic dosing, indications for treatment or referral, and management options that often form the basis of Applicability content. The absence of Accomodation and Communicability information in guideline development manuals may not be surprising. Traditionally guidelines have often focused on questions relevant to clinicians, and when gaps in care emerged, the focus of quality improvement has been on changing clinician behaviour. Recognition of the need to consider a broader array of factors that influence guideline use and impact, and the role of guidelines as tools to direct quality improvement in this broader context is relatively recent [8-10,21]. Similarly, the role of patients in the patient-provider dyad, clinical decision making, and even guideline development is evolving [31]. In keeping with the fact that new paradigms are emerging, our previous work found that 50% of the guidelines we examined contained Communicability information, and less than 50% contained Accomodation information [22].

The guideline enterprise is challenged to keep up with these changes and modify the traditional guideline development and implementation strategy. This may require consideration of different knowledge sources and evidence, involvement of different types of experts in the guideline development and implementation process either in staged fashion or as multidisciplinary or interprofessional teams, and partnerships between those who synthesize and interpret clinical effectiveness data and those who plan and/or undertake implementation. We know that guideline implementation is complex, and many developers lack the mandate and/or resources to implement the guidelines they produce [32,33], so an overarching question that must be addressed is who bears responsibility for implementing guidelines, what resources are required to support implementation as conceived in this emerging, broader paradigm, and who will provide those resources.

Interpretation of these findings may be confounded by several issues. Not all existing guideline manuals were examined. We instead relied on the comprehensive search strategy applied by another research team to identify our sample. They chose to examine six manuals that were produced by major international guideline development organizations, and of a general nature and therefore of broad relevance. We did search for and examine newer versions of those manuals if they were available. We also ran an updated search for other general guideline development manuals, and found only one published by the Canadian Medical Association [34]. For consistency and comparative purposes, we examined only the six manuals that were included in the previous study but brief review of the CMA manual revealed that it consists of 34 pages, of which one eleven-page chapter on implementation partially addresses Implementation and a three-page chapter partially addresses Evaluation. Instructions reflecting the remaining implementability domains and elements is lacking, therefore this manual too provides guideline developers with little instruction for preparing implementation advice.

Our implementability framework, while formulated based on expressed needs among various types of guideline users and studies positively associating these features with actual guideline use, has not been thoroughly validated. We have yet to conduct experimental studies that would test whether inclusion of information or tools reflecting these implementability domains leads to guideline implementation, use, and beneficial healthcare outcomes. We also need to assess how different types of users (clinicians, managers, policy makers) would interpret and use implementability tools. First, however, we need to develop implementability tools because our most recent analysis of guidelines (not yet published) found that few contained such tools. In advance of such development, we are further validating the framework with input from international guideline developers, implementers, and researchers. Despite the need for further validation, our framework offers a more comprehensive way to evaluate implementation instructions in guideline development manuals that reflects the emerging, more complex multi-step paradigm of guideline implementation. Therefore, this study provides a more detailed evaluation than previously published on whether differences in intrinsic qualities of guidelines may be due to differences in the content of development manuals, and more precisely how manuals could be refined to facilitate the development of guidelines that provide users with implementation advice. These findings can be used by those who developed the manuals to consider expanding their content. It can also be used by guideline developers as they plan the content and implementation of their guidelines so that the two are integrated.

Another limitation—not necessarily of our study, but in the evidence on guideline development and implementation—is the lack of criteria by which to assess the quality or actionability of instructions for developing guidelines or guideline implementation advice [35]. By using our more comprehensive implementability framework, we were able to comment on the completeness of the instructions. To judge the quality of the content of the instructions would require development of criteria, probably through expert consensus because little evidence is available, and then more detailed analysis of the content, both of which were beyond the resources available for the study described here. In ongoing research, we are searching for and examining the format and content of guidelines or adjunct products reflecting Accommodation, Implementation, and Evaluation to create ideal templates for tools offering implementation advice that will be vetted by both guideline developers and users.

In conclusion, most manuals that direct guideline development lack complete information about incorporating implementation advice for elements considered important by health professionals and associated with guideline use. These findings can be used by those who developed the manuals to consider expanding their content in these domains. It can also be used by guideline developers as they plan the content and implementation of their guidelines so that the two are integrated. However, to embrace the emerging expanded paradigm of guideline development and implementation, new approaches may need to be considered, including use of different knowledge sources and evidence, involvement of different types of experts, and partnerships between those who synthesize and interpret clinical effectiveness data and those who plan and/or undertake implementation. Who bears responsibility for implementing guidelines, what resources are required to support implementation, and who will provide those resources must also be considered. Use of guidelines might be improved if they included implementation advice, but this must be evaluated through ongoing research.

## Competing interests

The authors declare that they have no competing interests.

## Authors’ contributions

ARG conceived the study and its design, acquired funding, and coordinated its conduct. MCB contributed to study design, and participated in data interpretation. All authors read and approved the final manuscript.
